# The role of nitric oxide in portal hypertension caused by extrahepatic portal vein obstruction

**DOI:** 10.4103/0971-9261.72433

**Published:** 2010

**Authors:** P. Goel, K. Srivastava, N. Das, V. Bhatnagar

**Affiliations:** Department of Pediatric Surgery, All India Institute of Medical Sciences, New Delhi – 110 029, India; 1 Department of Biochemistry, All India Institute of Medical Sciences, New Delhi – 110 029, India

**Keywords:** Extrahepatic portal venous obstruction, nitric oxide, portal hypertension, porto-systemic shunt

## Abstract

**Aims:**

Nitric oxide (NO) in peripheral venous blood has been shown to be elevated in experimental portal hypertension. This study aims to determine the serum NO levels in patients with extrahepatic portal venous hypertension (EHPVO) pre- and postoperatively and to analyze whether these can serve as a reliable and early indicator of shunt blockage or malfunction.

**Materials and Methods:**

During the period 2006–2010, 48 children with EHPVO were included in this prospective study; 40 underwent porto-systemic shunt and eight underwent splenectomy with devascularization. NO was evaluated preoperatively, 14 days after surgery, at 3 months and then 6 monthly thereafter. The median follow-up duration was 21 months. Shunt patency was confirmed with Doppler and computed tomography portography. Forty-eight age-matched children with hypospadias served as controls.

**Results:**

NO was higher in EHPVO patients as compared with controls (43.16 ± 16.27 vs. 5.76 ± 2.62 *μ*mol/l) (*P* = 0.0001). There was a significant decline in the NO levels (4.64 ± 3.18 *μ*mol/l) following shunt surgery (*P* = 0.0001), and it continued to remain low till the shunt was patent. A shunt block was indicated by rising NO levels in all five patients. The devascularization group also demonstrated a significant decline in the NO levels (27.06 ± 3.56 *μ*mol/l) (*P* = 0.002), but it was less as compared with the shunted patients. The decline in the portal pressure after shunt surgery was found to correlate with the decline in the serum NO levels.

**Conclusions:**

The blood levels of NO can be used in the diagnosis of portal hypertension, and are useful for monitoring the patency of the shunt.

## INTRODUCTION

Definitive management of extrahepatic portal venous obstruction (EHPVO) involves creation of a porto-systemic shunt to decompress the portal venous system. Children are at a high risk of shunt blockage due to the small caliber of the veins; the reported shunt patency rates from centers experienced with complex vascular reconstruction of the portal system vary from 83 to 100%.[1,2] The postoperative follow-up of these patients, therefore, requires assessment of the shunt patency at regular intervals. There is a small subgroup of patients in whom shunt surgery is not possible, and they are provided symptomatic relief by splenectomy and devascularization of the gastro-esophageal junction.

The splanchnic and systemic circulations experience increased cardiac output and hyperdynamic circulation in portal hypertension.[3] This has been attributed to the relative excess in regional nitric oxide (NO) generation, which is endothelium mediated and dependant on endothelial nitric oxide synthase (eNOS).[4,5]

The aims of this study were to determine the levels of NO in the peripheral venous blood of the patients of EHPVO pre- and postoperatively and also to determine whether these can serve as a reliable and early indicator of shunt blockage or malfunction. A similar study has not been reported earlier.

## MATERIALS AND METHODS

All the patients of EHPVO who underwent surgical management during July 2006 to January 2010 under the care of a single surgeon were included in this prospective study. Appropriate approval from the Institute’s Ethics Committee and parental consent were obtained. Age-matched patients of hypospadias formed the control group.

Forty-eight patients were included in the study. The age varied from 4 years to 14 years, with a mean of 9.84 (± 3.13) years. There were 36 boys and 12 girls. Forty patients underwent porto-systemic surgery (lieno-renal shunt in 36, inferior mesocaval shunt in two, meso-caval interposition shunt in one, and the right gastro-epiploic vein was shunted to the renal vein in one patient). Eight patients underwent splenectomy and devascularization of the gastro-esophageal junction. The postoperative follow-up varied from 1 month to 39 months, with a mean follow-up of 21.08 (± 10.65) months. The median follow-up duration was 21 months.

The patients were evaluated for blood levels of NO preoperatively, 14 days after surgery, 3 months after surgery and at a gap of every 6 months subsequently. The shunt patency was confirmed with a simultaneous Doppler evaluation. A blocked shunt was confirmed by a computed tomography (CT) portogram. The control group was evaluated for NO only once.

Intraoperatively, a venous tributary of the gastro-epiploic arcade was cannulated with a 20-gauge intravenous cannula and connected to the pressure transducer for measurement of the portal pressure before and after splenectomy and the creation of the shunt.

### Biochemical Analysis

Peripheral venous blood was collected in heparinized tubes, centrifuged (4°C, 3000 rpm, 10 min) and preserved at -70°C. During analysis, nitrate was reduced to nitrite by nitrate reductase and then the plasma level of NO was quantified spectrophotometrically (540 nm) by the Greiss reaction.

### Statistical Analysis

The statistical analysis was performed using SPSS 14.0 and Stata 9.0. Data were presented as number (%) or mean (± SD)/median (range) as appropriate. The levels of NO were compared between EHPVO patients and the controls using the paired *t*-test. The serial levels of NO were also compared with the paired *t*-test. However, the levels between the patients who underwent shunt surgery and those who underwent devascularization were compared using the Mann–Whitney U test. The correlation between the postoperative drop in the NO levels and portal pressures was calculated using the Spearman rank correlation coefficient. A *P*-value <0.05 was considered statistically significant.

## RESULTS

### Nitric Oxide Levels

The mean level of NO was 43.16 ± 16.27 *μ*mol/l in the patients of EHPVO and 5.76 ± 2.62 *μ*mol/l in the control population. The difference between the two values is significant (*P* = 0.0001). Following shunt surgery, there is a significant decline in the levels of NO.

The levels of NO after the shunt surgery are comparable to the levels of NO in the control population [Table 1].

**Table 1:** Paired *t*-test comparing the nitric oxide levels in the EHPVO patients preoperatively and postoperatively vis-à-vis the controls

	NO levels (μmol/liter)		
	EHPVO	*P*-value	
	patients		
Preoperative NO levels	43.16 ± 16.27	Preop vs. post	Postop vs. ctrl
		op	
Postoperative (Day 14) NO levels	4.64 ± 3.18	0.0001	0.062

After a successful shunt surgery, the NO levels continue to remain low till the patency of the shunt is maintained [Table 2].

**Table 2:** Paired t-test comparing the NO levels in the patients of EHPVO in follow-up

Time of postop NO estimation	NO levels (μmol/l)					
	Patent shunt		Blocked shunt group		Devascularization group	
	No. of patients	Mean ± SD	No. of patients	Mean ± SD	No. of patients	Mean ± SD
Day 14	37	4.64 ± 3.18	5	16.84 ± 29.20	8	49.14 ± 12.77
3 months	37	4.38 ± 2.63	4	5.76 ± 4.92	7	27.06 ± 3.56
6 months	34	4.41 ± 2.23	4	13.03 ± 3.22	4	24.23 ± 5.91
1 year	32	5.25 ± 4.69	4	23.97 ± 13.49	1	20.00
1.5 years	24	4.21 ± 2.18	4	24.70 ± 9.94		
2 years	22	4.77 ± 2.35	3	28.55 ± 14.47		
2.5 years	10	3.74 ± 1.91	2	32.7 ± 5.37		
3 years	7	3.53 ± 2.35	1	42.50		

**Figure 1:**
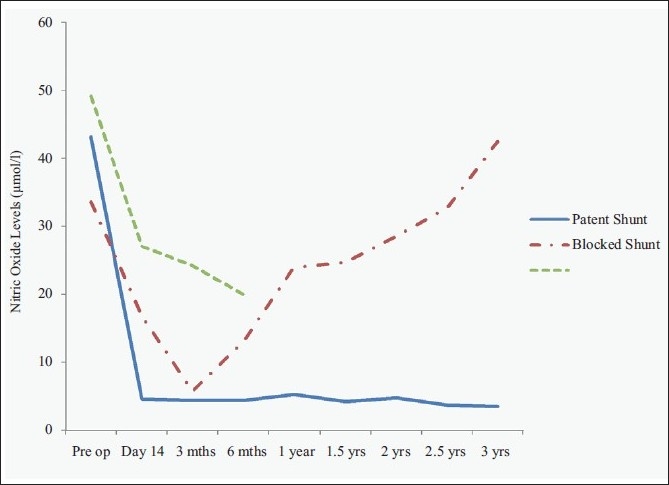
Comparative depiction of the nitric oxide levels (μ moles/litre) in patients of extrahepatic portal venous hypertension

Five patients had a blocked shunt in the postoperative period. The first patient (lieno-renal shunt) had a shunt block at 6 months after surgery. The second patient (inferior meso-caval shunt) was re-explored at 3 months post shunt surgery for adhesive intestinal obstruction. At that time, the shunt was evaluated and found to be patent. However, at 6 months of follow-up, the shunt was blocked. Two patients (lieno-renal shunt) had a shunt block on postoperative day 14. In all these patients, the NO levels had shown an initial decline after the shunt surgery and a subsequent rise [Table 2]. The fifth patient (mesocaval interposition shunt) never showed a decline in the NO levels, and the shunt was found to be blocked on Doppler evaluation in the immediate postoperative period. The shunt was recanalized by an interventional radiologist; however, the child died of sepsis and disseminated intravascular coagulation. The trend of NO in all these patients has been depicted in Figure 1 (collectively) and 2 (individually).

**Figure 2:**
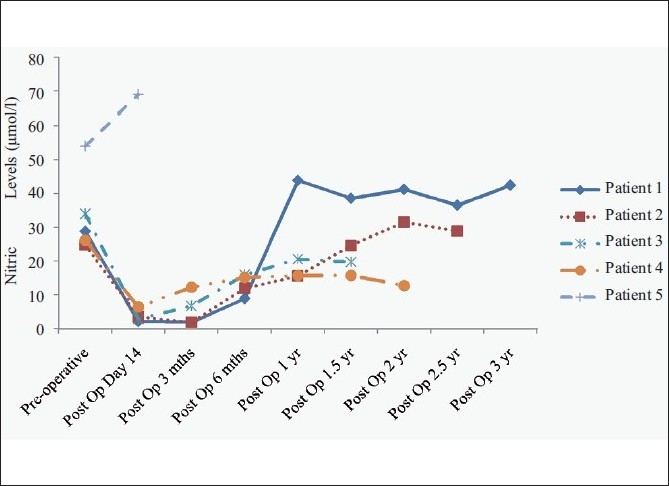
Serial nitric oxide levels (μ moles/litre) in patients of extrahepatic portal venous hypertension who underwent shunt surgery and later developed a shunt block

The patients who underwent devascularization also showed a significant decline in the NO levels [Table 2]. However, the statistical analysis has revealed that the decline was higher in case of patients who underwent shunt surgery. The mean levels of NO after devascularization are higher as compared with those in patients who underwent a shunt surgery instead. Post splenectomy and devascularization, although the serum levels of NO come down, they are still not comparable to the NO levels in the control population [Table 3].

**Table 3:** Statistical analysis comparing the NO levels in patients who underwent devascularization with the controls and with the shunted patients

	Test	*P*-value
Preop vs. postop Day 14 (Devasc.)	Paired *t*	0.002
Controls vs. postop Day 14 (Devasc.)	Paired *t*	0.0001
Shunt vs. devasc. on postop Day 14	Mann–Whitney	0.0001

**Figure 3:**
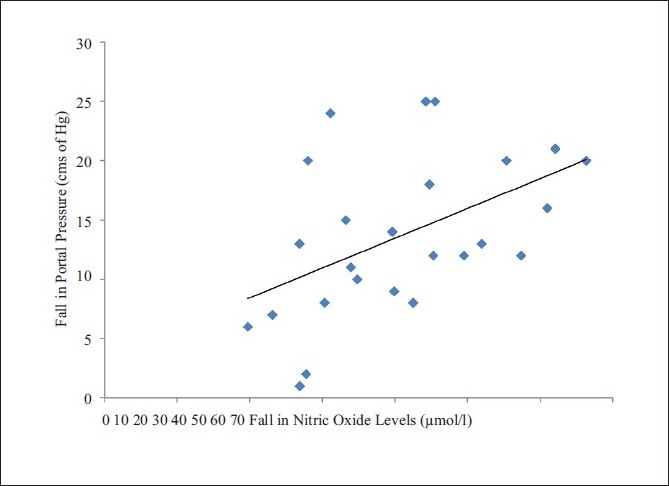
Correlation between fall in nitric oxide levels and fall in portal pressure before and after shunt surgery

The mean preoperative portal pressure was 34.19 ± 5.90 mmHg, which fell to 20.88 ±± 6.51 mmHg after splenectomy and creation of the porto-systemic shunt. The correlation between the drop in the blood levels of NO and the drop in the portal pressures [Figure 3] was found to be statistically significant (*P* = 0.002).

**Figure 4:**
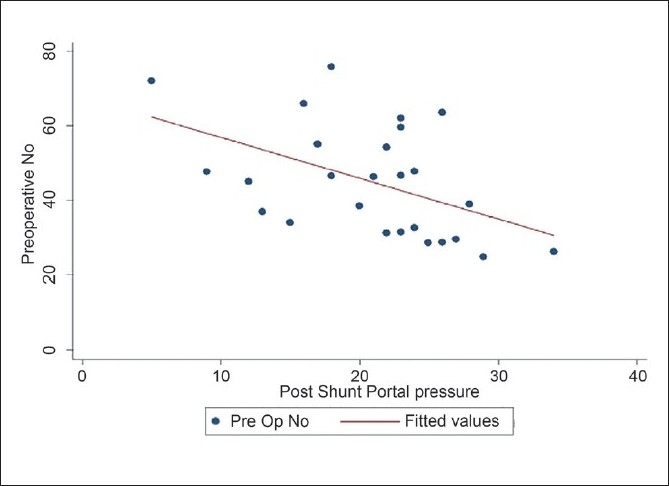
Correlation between the preoperative nitric oxide levels and the portal pressures after shunt surgery

No correlation could be established between the preoperative levels of NO and the preshunt portal pressures. However, there was a negative correlation between the preoperative levels of NO and the postshunt portal pressure, which was statistically significant (*P* = 0.010) [Figure 4].

## DISCUSSION

The splanchnic and systemic circulations experience increased cardiac output and hyperdynamic circulation in portal hypertension.[3] This has been attributed to the relative excess in regional NO generation, which is endothelium mediated and eNOS dependant.[4,5] Mesenteric arteries of rats with portal hypertension exhibit markedly enhanced eNOS protein expression, which precedes the development of the hyperdynamic splanchnic circulation.[6–8] Experimental ligation of the portal vein in rats leads to a significant increase in the serum NO levels and NOS activity at 24 h compared with the controls. The rising levels of NO peak at 48 h, and remain constant thereafter.[9]

Based on these observations, it was hypothesized that the levels of NO in the patients of EHPVO should be elevated. The study has shown that the levels of NO are elevated in the peripheral venous blood of patients with EHPVO. After surgical correction, the levels of NO have shown a decline. The levels of NO become comparable to the controls after porto- systemic shunt surgery.

The levels of NO also come down after splenectomy and devascularisation, which suggests that the procedure is effective in lowering the portal pressure. However, in these patients, NO levels are still higher than the age-matched controls. This is possibly due to the portal pressure still remaining higher than normal after devascularization and an equilibrium being reached with some collaterals remaining open. On the other hand, in the case of porto-systemic shunting, the portal pressure normalized and the collaterals possibly obliterate spontaneously. With blockage of the shunt, the portal pressures rise dramatically in the absence of the collaterals. This is reflected by the rise in the NO levels.

The authenticity of NO is further confirmed by correlating it with the portal pressures measured intraoperatively. High levels of NO are associated with high levels of portal pressure. The magnitude of fall in the levels of NO with the shunt surgery has been found to correlate with the magnitude of fall in the portal pressure.

Children undergoing porto-systemic shunting are at a high risk of shunt blockage due to the small caliber of the veins. They need to be evaluated for the patency of the shunt regularly, using ultrasound Doppler as the first line of investigation for this purpose. However, its usefulness is limited by its operator dependence, poor reproducibility and the problem associated with ascites or overlying gas-filled bowel loops. Medical personnel other than the radiologist find it difficult to interpret the images. CT portogram was used to confirm the shunt block in all the patients. Its use is limited by its cost, availability of equipment and technical expertise, radiation hazards, need for intravenous contrast material, artefacts from surgical clips, technically poor images due to motion artefacts and inability to quantify the venous flow across the anastomosis. MR imaging is also limited by its availability, cost, occasionally artefacts and the time taken.

During the follow-up of these patients, the patency of the porto-systemic shunt has been found to correlate with the NO levels in our study. NO shows a steep rise after the blockage of the shunt, and continues to rise for a few months initially, thereby showing a plateau. In this way, it has served its role as a marker of shunt patency. Estimation of NO is a simple and relatively inexpensive laboratory procedure; the only requirement is 2 ml or perhaps even lesser amount of peripheral venous blood from the patient. No special equipment or manpower training is required.

## CONCLUSIONS

The blood levels of NO could be utilized as a marker of portal hypertension and may also be used as a marker for the patency of the shunt in the follow-up of these patients.
